# Insights into Functionalization of Metal-Organic Frameworks Using *In Situ* NMR Spectroscopy

**DOI:** 10.1038/s41598-018-35842-1

**Published:** 2018-12-03

**Authors:** Ning Yuan, Tamara L. Church, Erik G. Brandt, Niklas Hedin, Xiaodong Zou, Diana Bernin

**Affiliations:** 1 Department of Materials and Environmental Chemistry, Stockholm University, SE-106 91 Stockholm, Sweden; 2 Department of Chemistry and Chemical Engineering, Chalmers University, SE-412 96 Gothenburg, Sweden; 3 Department of Molecular Sciences, Swedish University of Agricultural Sciences, SE-750 07 Uppsala, Sweden

## Abstract

Postsynthetic reactions of metal-organic frameworks (MOFs) are versatile tools for producing functional materials, but the methods of evaluating these reactions are cumbersome and destructive. Here we demonstrate and validate the use of *in situ* NMR spectroscopy of species in the liquid state to examine solvent-assisted ligand exchange (SALE) and postsynthetic modification (PSM) reactions of metal-organic frameworks. This technique allows functionalization to be monitored over time without decomposing the product for analysis, which simplifies reaction screening. In the case of SALE, both the added ligand and the ligand leaving the framework can be observed. We demonstrate this *in situ* method by examining SALE and PSM reactions of the robust zirconium MOF UiO-67 as well as SALE with the aluminum MOF DUT-5. *In situ* NMR spectroscopy provided insights into the reactions studied, and we expect that future studies using this method will permit the examination of a variety of MOF–solute reactions.

## Introduction

Metal-organic frameworks (MOFs) are crystalline porous materials that have applications including gas storage/separation, catalysis, and molecular sensing^1–5^; thus, there is a demand for postsynthetic functionalizations to tailor the physicochemical properties of MOFs^6^. Functional groups can be introduced into a MOF via the postsynthetic modification of its linkers^3,7^. Alternatively, in solvent-assisted linker exchange (SALE), framework linkers are replaced with functionalized analogues under mild reaction conditions in order to introduce functional groups or alter the pore size while retaining the crystallinity of the MOF^8–11^. Although SALE is extensively used, the underlying mechanisms are still poorly understood^6^, meaning that a time-consuming optimization of synthesis conditions^3^ is required for each new modification. The fraction of functionalized linkers incorporated during SALE has been, until now, estimated from liquid-state ^1^H NMR spectroscopy of the solution produced by digesting the functionalized MOF in a strong corrosive acid (e.g. HF(*aq*)) or base^12–17^. *In situ* methods could enable direct measurements, avoiding this destructive step. Other information that can be obtained from liquid-state ^1^H NMR includes the pH^18^ of a reaction mixture and the formation of unexpected compounds. The hydrothermal synthesis of aluminum-based metal-organic frameworks has been studied using *in situ* NMR in specialized equipment designed to withstand high temperatures and pressures;^19^ however, the need for specialized equipment could restrict the use of *in situ* NMR in the case of hydrothermal synthesis^20^.

Here, we investigated the use of *in situ* ^1^H NMR to monitor reactions of MOFs with dissolved species, and thus to derive time-resolved information about the functionalization of MOF. Jeong *et al*. have recently used NMR and *in situ* NMR as part of a study that examined the exchange of solvents coordinated to metal centres in a MOF^21–23^, and we here extend the *in situ* approach to several types of MOF–solution reactions, and investigate whether it is consistent with *ex situ* methods.

## Results and Discussion

We first applied an *in situ* approach to examine SALE in the robust zirconium MOF UiO-67 (UiO: University of Oslo)^24^. Fei and Cohen^13^ have used SALE to replace the biphenyl 4,4′-dicarboxylate (bpdc) ligand in the original MOF (here labeled UiO-67-bpdc) with 2,2′-bipyridine 4,4′-dicarboxylate (bpydc), which can support catalytic metal species^25–33^. Functionalization of UiO-67 has been studied using *ex situ* measurements^27^, as has the interaction of UiO-67 with various solvents and chemicals^34^. Prior to monitoring SALE in UiO-67 using *in situ* ^1^H NMR, we first considered some practical issues. In order to obtain quantitative information about dissolved species, it is essential to avoid relaxation-weighted NMR intensities; thus, the repetition delay was optimized prior to gathering reaction data. Further, in order to minimize the effect of mass transfer on reactions in the NMR tube, the MOF was ground before use. Finally, the multiphase SALE reaction can only be followed by *in situ* ^1^H NMR if the exchanging linker is present in the liquid state, rather than adsorbed on or in the MOF. To test whether this was the case, we measured the room-temperature *ex situ* ^1^H NMR spectrum of a solution of bpdc in deuterated dimethylsulfoxide (DMSO-*d*_6_) with an internal standard, before and after the addition of 2 mg UiO-67-bpdc. The measured intensity of bpdc fell by ~9% after the MOF was added, indicating that only a small amount of linker was adsorbed under these conditions; even less adsorption is expected at the higher temperatures used in SALE (vide infra).

**Figure 1:**
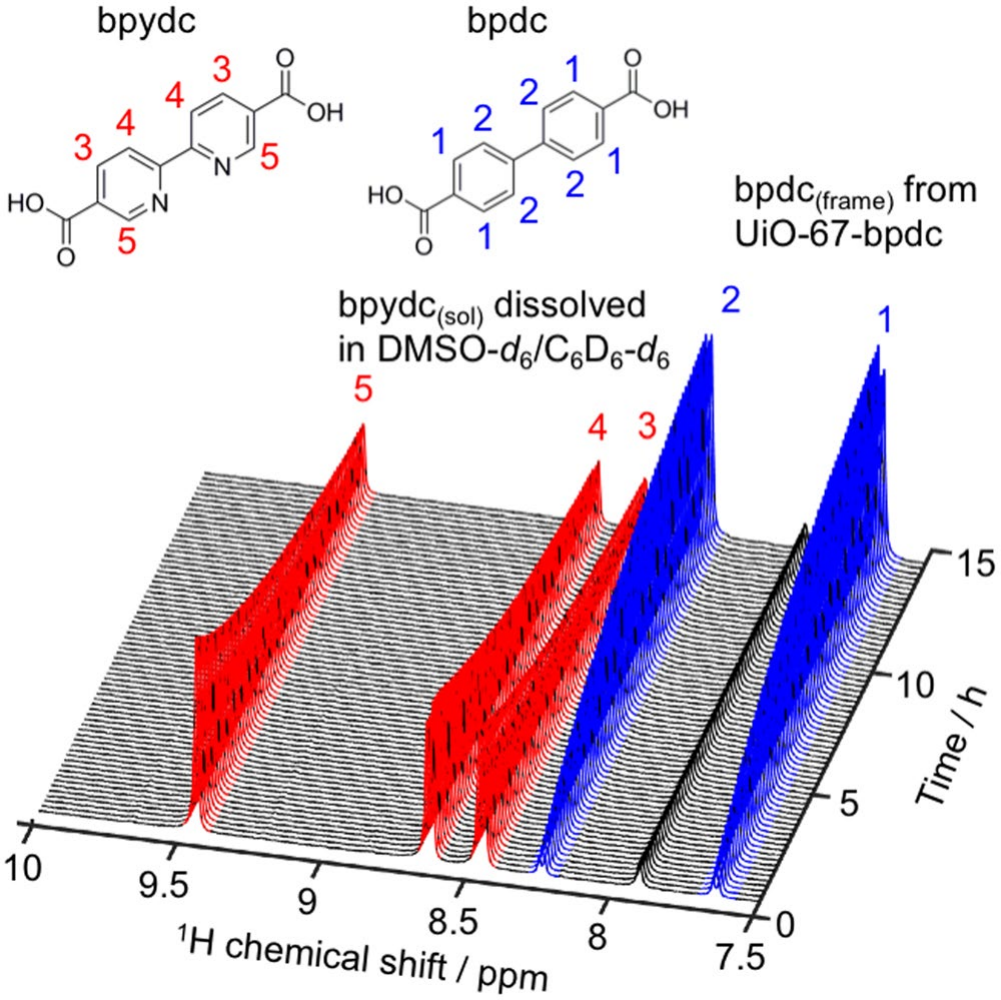
Molecular structures of the protonated linkers bpydc and bpdc with ^1^H NMR peak assignments (top), and stacked ^1^H NMR spectra versus time (bottom) for the solvent-assisted linker exchange reaction of UiO-67-bpdc with bpydc_(sol)_ in 37 mol% DMSO-*d*_6_ in C_6_D_6_ at 57 °C.

Having confirmed that bpdc is observed by *ex situ* ^1^H NMR in the presence of UiO-67, we monitored SALE between UiO-67-bpdc and bpydc in real time using *in situ* ^1^H NMR. In the following discussion, we do not distinguish the neutral and the singly and doubly deprotonated forms of the ligands; rather, we use the extension _(sol)_ to indicate any form of the ligand that was initially present in solution, and _(frame)_ to indicate any form of the linker that was initially present in the MOF. In each reaction, ground UiO-67-bpdc was combined with a solution of bpydc_(sol)_ in an NMR tube to give a mixture with linker_(frame)_:linker_(sol)_ in the range 0.5–1.5. The NMR tube was immediately inserted into the NMR probe, which had been preheated to 57 °C. *In situ* ^1^H NMR spectra collected throughout the reaction showed the disappearance of bpydc_(sol)_ as it was either adsorbed onto or incorporated into the MOF, concurrently with the appearance of bpdc_(frame)_ in solution (Fig. 1). As the sample remained in the NMR spectrometer for the duration of the experiment, no standard solution was necessary. No peaks associated with acetic acid, the modulator used to prepare UiO-67-bpdc (see Supporting Information, section S3), were detected.

**Figure 2:**
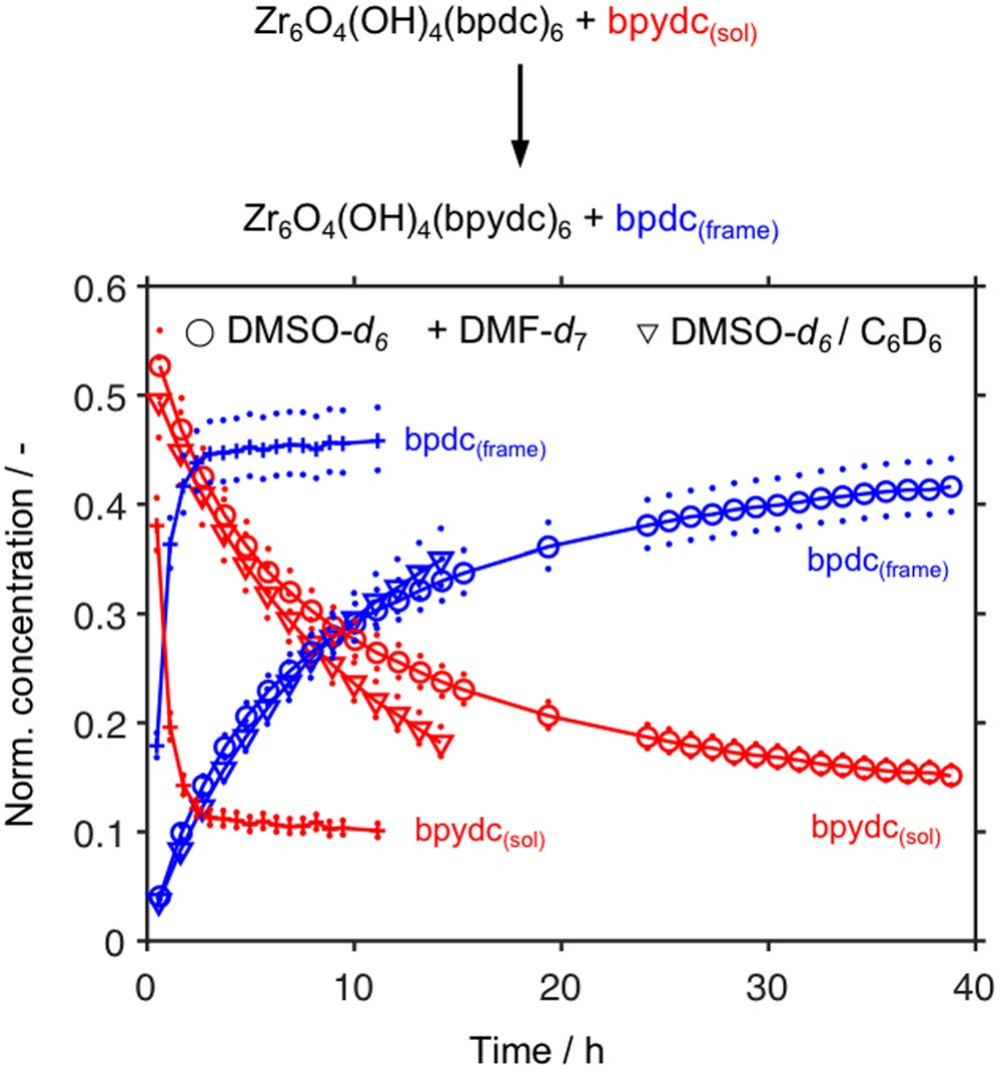
The normalized concentrations of linkers (see Exp. Section) in the liquid state during the reaction of UiO-67-bpdc and bpydc_(sol)_ as functions of time in three solvent systems. Reactions were performed at 57 °C and monitored using *in situ* ^1^H NMR spectroscopy. Dots represent the spread of the normalized concentrations calculated from the mass of MOFs present and the uncertainty of the balance (±0.1 mg). For clarity, only every 10^th^ data point is shown.

The rate of linker exchange between UiO-67-bpdc and bpydc_(sol)_ depended strongly on solvent (Fig. 2) and was slowest in DMSO-*d*_6_. The reaction was slightly faster in a mixture of DMSO-*d*_6_ and C_6_D_6_ (37 mol% DMSO-*d*_6_) than in DMSO-*d*_6_ alone. This observation could be partially due to the comparably higher viscosity of DMSO-*d*_6_, which is estimated to permit linker diffusion at just over half the rate that is possible in the DMSO-*d*_6_/C_6_D_6_ mixture (Supporting Information); however, DMSO-*d*_6_ also clearly produces a lower reaction rate. This solvent cannot be omitted entirely as neither bpdc nor bpydc is soluble in C_6_D_6_. SALE between UiO-67-bpdc and bpydc_(sol)_ was fastest in deuterated dimethylformamide, (DMF-*d*_7_), and the reproducibility of the *in situ* ^1^H NMR measurement was confirmed in this solvent (Fig. S5). In all cases, the disappearance of bpydc_(sol)_ from solution in the presence of UiO-67-bpdc followed pseudo-first-order kinetics (Fig. S6).

Although the substitution of bpydc_(sol)_ into UiO-67-bpdc was much faster in DMF-*d*_7_ than in DMSO-*d*_6_, both reactions eventually reached a steady degree of exchange (40–45% under these conditions). This illustrates an important point for SALE; if sufficient time is provided, the extent of exchange can be determined by thermodynamic factors, i.e. the relative stability of the linkers in the MOF and in the solution. DMF-*d*_7_ and DMSO-*d*_6_ are both polar aprotic solvents, and thus it is reasonable that similar degrees of exchange are reached from the same starting point in both solvents. The degree of exchange could be different for other linker pairs and in other solvents, and *in situ* NMR offers a convenient method to examine the multiple sets of reaction conditions using small amounts of material. Martín–Matute, Zou, and co-workers have shown that undesirable reactions can occur between functionalized MOFs and solutions upon extended contact^27^, so information regarding the maximum useful time for SALE is crucial for optimizing the functionalization of the MOF. For example, in the present case, continuing to heat UiO-67-bpdc and bpydc_(sol)_ in DMSO-*d*_6_ for more than 40 h would not result in a large increase in the degree of exchange; however, a separate *in situ* experiment (Fig. S7) demonstrated that the degree of exchange could be raised to 56% by raising the bpydc_(sol)_: bpdc_(frame)_ ratio from 0.57 to 1.4.

**Figure 3:**
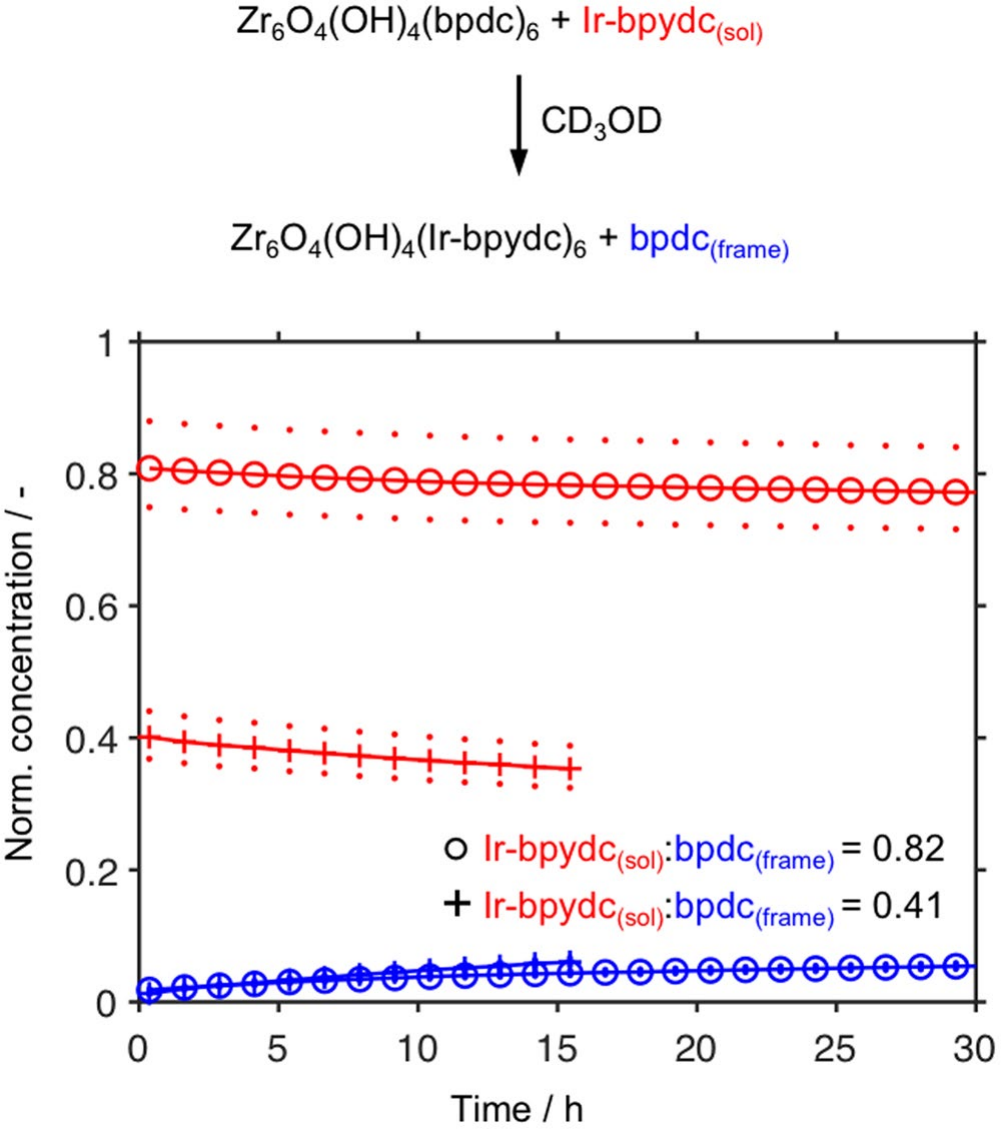
Normalized concentrations of linkers (see Exp. Details) in the liquid state as functions of time during the reaction of UiO-67-bpdc with [Cp*Ir(bpydc)Cl]^+^ (Ir-bpydc_(sol)_) in CD_3_OD at 57 °C. Dots represent the spread of the normalized concentrations calculated from the mass of MOF present and the uncertainty of the balance (0.1 mg). For clarity, only every 12^th^ data point is shown.

SALE can also be relevant in the construction of MOFs bearing catalytically active complexes; in fact, such MOFs can be produced via several routes. For example, iridium-containing MOFs have been synthesized directly using iridium complexes with ligands bearing carboxylate groups as linkers^35,36^, but have also been synthesized using a mixture of linkers^25,26^ or via the postsynthetic modification of a MOF^36,37^. Martín–Matute, Zou, and co-workers have observed the undesired demetallation of Ir-containing MOFs (a side reaction that is easily detected by liquid-state ^1^H NMR) under some reaction conditions^27^. We therefore investigated the exchange of bpdc_(frame)_ in UiO-67-bpdc with [Cp*Ir(bpydc)(Cl)]Cl (we hereafter denote [Cp*Ir(bpydc)Cl]^+^, as well as its analogues that are singly and doubly deprotonated at the carboxylic acids, collectively as Ir-bpydc) in CD_3_OD (Fig. 3). We found that 6.4 and 5.6% of the bpdc_(frame)_ were replaced with functionalized linker for starting linker_(sol)_:linker_(frame)_ ratios of 0.82 and 0.41, respectively. No additional ^1^H NMR peaks consistent with Cp*Ir-containing species were observed over the course of the 30-h reaction, which indicates that no demetallation occurred. Further, no exchange was observed between UiO-67-bpdc and Ir-bpydc_(sol)_ in DMSO-*d*_6_ or ethanol-*d*_6_.

**Figure 4:**
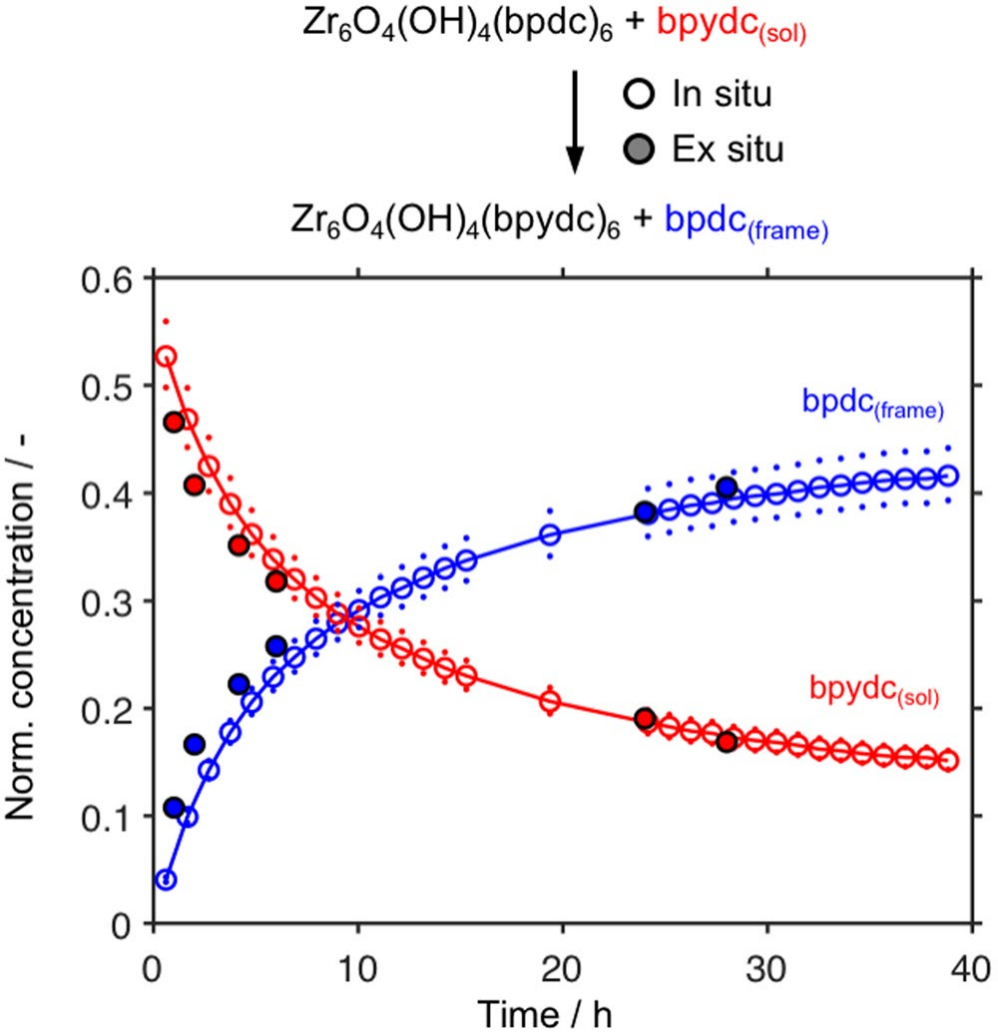
Normalized concentrations of linkers (see Exp. Details) in the liquid state of SALE as functions of time, as calculated from liquid-state ^1^H NMR spectra. Reactions were performed in DMSO-*d*_6_ at 57 °C. Dots represent the spread of the normalized concentrations calculated from the mass of MOF present and the uncertainty of the balance (0.1 mg). For clarity, only every 12^th^ data point is shown in the *in situ* data series.

In order to compare the results obtained from reactions performed in NMR tubes to the larger-scale methods conventionally used for SALE, we performed two types of *ex situ* experiments. First, the reaction of UiO-67-bpdc with bpydc_(sol)_ in DMSO-*d*_6_ was scaled up sevenfold and performed in vials. The mixture was stirred with magnetic bars. Aliquots were removed from the reaction mixture and analyzed by ^1^H NMR spectroscopy (Fig. 4, filled symbols). The *ex situ* and *in situ* measurements produced very similar results. In two experiments scaled up to 50–60 mg MOF, UiO-67-bpdc was combined with bpydc_(sol)_ in non-deuterated DMSO and DMF for 24.5 and 18 h, respectively. The products were filtered and washed with ethanol, and the resulting solids were examined using powder X-ray diffraction and N_2_ sorption (Supporting Information, Figs S2 and S4) to confirm that the crystalline and porous structure of the MOF were retained. The solids were then examined using solid-state ^1^H NMR spectroscopy (Supporting Information, Figs S9 and S10). The material exchanged in DMSO contained 41% bpydc, the same fraction calculated from the *in situ* experiment (Fig. 2, circles). In DMF, the product was 45 and 53% bpydc when observed *ex situ* and calculated from *in situ* data, respectively. The minor discrepancy between the values *in* and *ex situ* in DMF likely occurs because the ^1^H NMR peaks for bpdc overlap with that for the aldehyde proton in DMF, complicating their integration. Thus overall, monitoring the reaction of UiO-67-bpdc with bpydc_(sol)_ using *in situ* ^1^H NMR proved a convenient method to obtain data that was consistent with observations made on larger scales.

Although SALE is most commonly performed with the aim of replacing a less-functionalized linker with a functionalized one, the converse reaction can also be examined using *in situ* ^1^H NMR. However, we found the reaction of UiO-67-bpydc with bpdc_(sol)_ in DMSO-*d*_6_ to give varied results (Fig. S8), regardless of whether the reaction was performed in the NMR spectrometer or *ex situ* in vials. The mechanism of SALE is complex for at least some systems^16^, and it is clear that this reaction requires further study.

**Figure 5:**
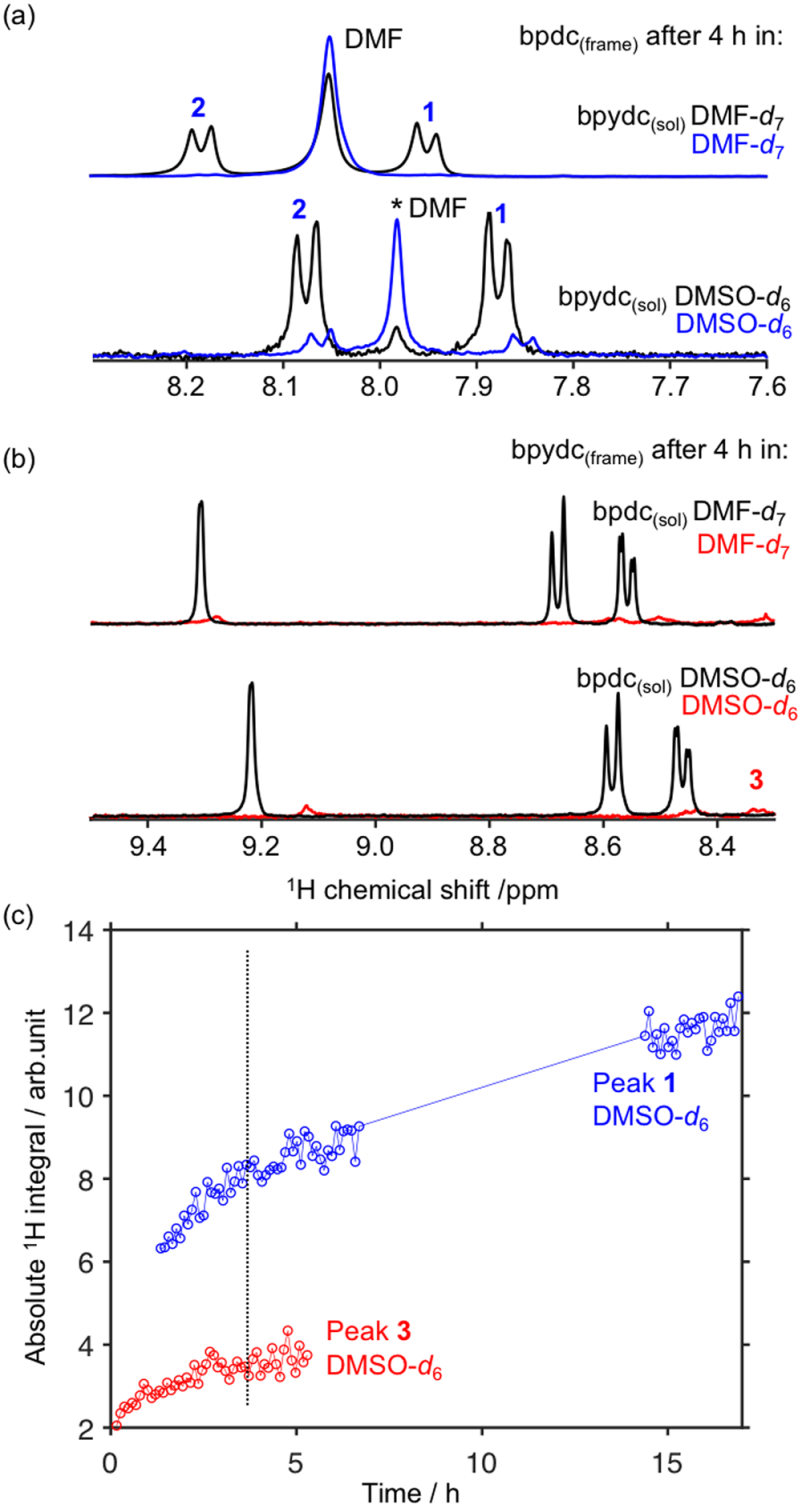
*In situ* ^1^H NMR spectra recorded after heating (**a**) UiO-67-bpdc and (**b**) UiO-67-bpydc in solvent, with and without added ligand_(sol)_. The asterisk indicates an impurity derived from the synthesis of the MOF. (**c**) Absolute ^1^H integral for peaks 1 and 3 (see assignments in Fig. 1) versus time for dissolution of UiO-67-bpdc and UiO-67-bpydc in DMSO-*d*_6_.

The stability of UiO-67 in solvent has been the subject of literature discussion^34,38–40^, and the appearance of linker_(frame)_ from UiO-67 in a solvent was therefore examined by soaking UiO-67-bpdc or UiO-67-bpydc (the same amounts as used for SALE, Figs 2 and 3) in DMSO-*d*_6_, DMF-*d*_7_, or D_3_COD at 57 °C and monitoring the *in situ* ^1^H NMR spectra of the reaction (Fig. 5). Regardless of the starting MOF, only a small amount of linker_(frame)_ was released into DMF-*d*_7_ over the course of 4 h, in stark contrast to the rapid SALE observed in this solvent. The release of linker_(frame)_ was also much slower in DMSO-*d*_6_ when no linker_(sol)_ was present. However, when 1 μL acetic acid was added to the mixture of UiO-67-bpydc in DMF-*d*_7_ or DMSO-*d*_6_, bpydc_(frame)_ began to appear in solution (Fig. S11). Thus this *in situ* ^1^H NMR technique may also be of use in examining the formation of defects in MOFs, as defects can be introduced using solution-based chemistry^41^. Linker_(frame)_ did appear in solution when UiO-67-bpdc was soaked in CD_3_OD at 57 °C (Fig. S12), even without added acid, indicating that defects can be introduced into this MOF using solvent if the solvent and temperature are chosen accordingly.

**Figure 6:**
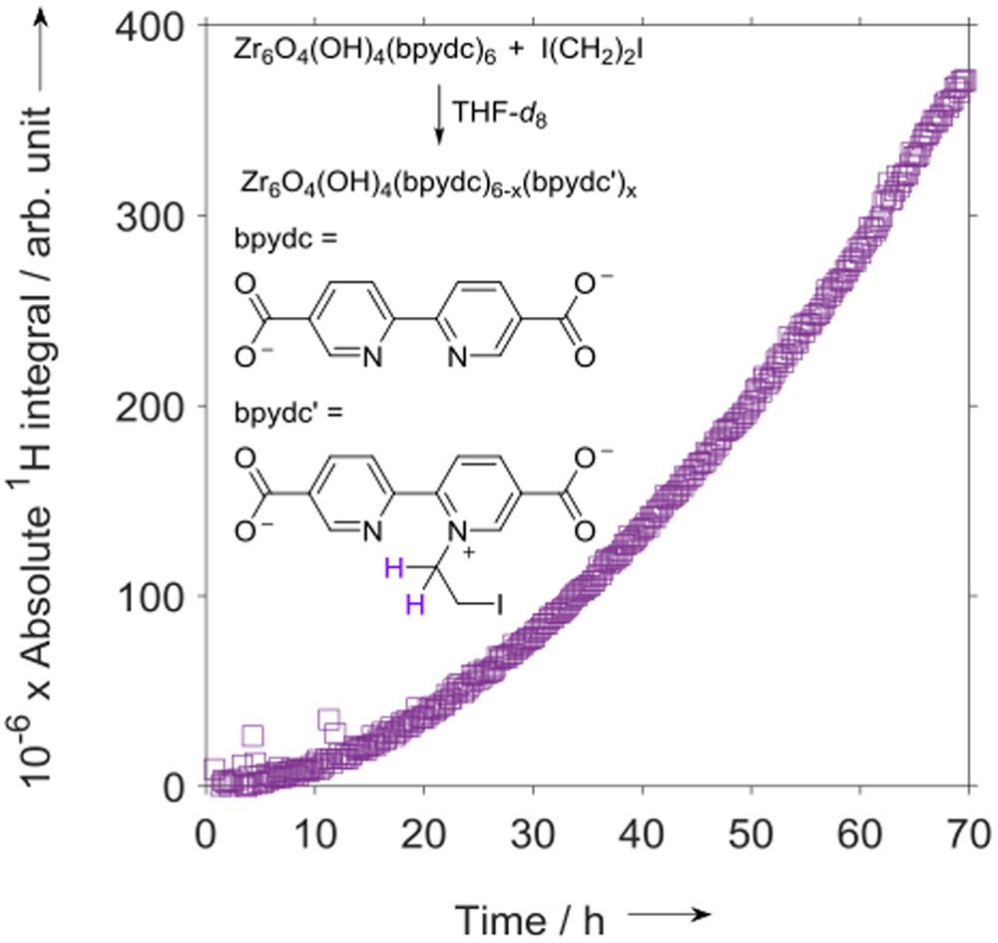
Absolute integral of the ^1^H NMR peak at a chemical shift of 5.3 ppm in spectra recorded *in situ* during the reaction of UiO-67-bpydc with I(CH_2_)_2_I in THF-*d*_8_ at 55 °C.

In addition to SALE, the *in situ* ^1^H NMR method described here should be useful for studying other reactions between MOFs and dissolved species, and we therefore examined the course of a PSM. Pyridinic N atoms in and pendant from MOF linkers have been alkylated using alkyl iodides^42–44^ or sulfonates^45^. We therefore followed the reaction of UiO-67-bpydc with I(CH_2_)_2_I in THF-*d*_8_ at 55 °C (Fig. 6).

In this case, the growth of a ^1^H NMR peak at a chemical shift of 5.3 ppm, which represents an –N^+^–CH_2_– moiety^46^, was monitored. Thus, a somewhat different strategy was used to follow this reaction than to study SALE (vide supra), in that the species monitored here was bound to the MOF. This particular species could be detected using liquid-state NMR because, as a pendant alkyl group, it has significant rotational freedom and hence is detectable. On the other hand, if the bifunctional I(CH_2_)_2_I formed a bridge between the two pyrdinic N atoms in a bpydc linker, the resulting liquid-state ^1^H NMR signal intensity would be expected to decay rapidly, resulting in a broad bump or a lack of signal. The intensity of the peak at 5.3 ppm was still increasing after 60 h when the sample was removed from the spectrometer. Only trace signals were observed in the aromatic region of the ^1^H NMR spectra, which is further evidence that the alkylpyridinium moieties were bound in the MOF and that very little bpydc_(frame)_ was released in THF at 55 °C, at least when I(CH_2_)_2_I was present. After the *in situ* PSM, the sample was filtered and the resulting yellowish solid washed with non-deuterated THF. The UV-vis spectrum of the solid (Fig. S13) featured a broad absorbance centered at approximately 400 nm, which confirmed the presence of the alkylpyridinium moiety^43^.

To our knowledge, there are no reports of SALE in the aluminum MOF DUT-5-bpdc. Our *in situ* ^1^H NMR method, which requires only 1–2 mg of MOF per trial, enabled us to straightforwardly assess the feasibility of this reaction under the conditions used for SALE in UiO-67. Hence, we monitored SALE to exchange bpydc_(sol)_ with bpdc_(frame)_ in DUT-5-bpdc. No reaction occurred in DMF-*d*_7_ or DMSO-*d*_6_, although the modulator used in the preparation of DUT-5-bpdc, benzoic acid, was observed in solution. This conclusion was reached with a minimal use of material.

## Conclusions

The presented studies of linker exchange and modification in MOFs demonstrate that *in situ* NMR is a versatile and straightforward tool to study the reactions of MOFs with solvents or dissolved species. Reaction conditions such as solvent, concentration and temperature are easily varied; thus, the method promises to greatly improve the efficiency with which conditions for MOF–solute reactions can be screened. We believe that this method could also be used to give valuable insights into defect formation and control as well as into postsynthetic modifications such as deprotection and insertion reactions. Detailed mechanistic investigations of other MOF–solution reactions using *in situ* ^1^H NMR measurements are underway.

### Experimental Section

#### *In situ*^1^H NMR spectroscopy

Proton (^1^H) nuclear magnetic resonance (NMR) experiments were recorded on a 9.4 T (^1^H Larmor frequency 400 MHz) Bruker Avance spectrometer equipped with a 5-mm double resonance broadband probe (BBI) optimized for ^1^H detection. The repetition time was set to 10 s and the acquisition time was 1.5 s. A single pulse experiment with a radiofrequency pulse of between 30–80° was employed to record the NMR signal. The spectral width was 37.4 ppm and the signal was accumulated 32 times. The recorded signal was processed with Matlab (Mathworks) using own scripts.

The number of moles of linkers in solution were normalised using the sum of the peak integrals of the linkers from the first recorded experiment and the known amount of linker_(sol)_. To account for the different linker_(sol)_:linker_(frame)_ ratios, the moles of linker were normalized by the known moles of the linker_(frame)_.

For further experimental details, see Supplementary Information.

## Electronic supplementary material

Supplementary information

## Data Availability

The datasets generated and analysed during the current study are available from the corresponding author on request. The data points in Figs 2–6 are obtained from the ^1^H NMR spectra displayed in Figs S15–23 the Supplementary Information.
