# Supplementary material for: Insights into Functionalization of Metal-Organic Frameworks Using In Situ NMR Spectroscopy
Source: Sci Rep. 2018 Dec 3;8:17530. doi: 10.1038/s41598-018-35842-1 (PMC6277383; doi:10.1038/s41598-018-35842-1)
Supplement: Supplementary file 1 — Supplementary information [file 41598_2018_35842_MOESM1_ESM.docx]

Supporting Information for

Insights into Functionalization of Metal-Organic Frameworks Using In Situ NMR Spectroscopy

Ning Yuan^‡[1]^, Tamara L. Church^‡[1]^, Erik G. Brandt^[1]^, Niklas Hedin^[1]^, Xiaodong Zou^[1]^, Diana Bernin^[1,2]^*

[1] Department of Materials and Environmental Chemistry, Stockholm University, SE-106 91 Stockholm, Sweden

[2] Department of Chemistry and Chemical Engineering, Chalmers University, SE-412 96 Göteborg

‡ These authors contributed equally to the work.

* To whom correspondence should be addressed. E-mail: [diana.bernin@chalmers.se](mailto:diana.bernin@chalmers.se)

**Table of contents:**

[S1. Materials 2](#_Toc527840149)

[S2. Methods 2](#_Toc527840150)

[NMR 2](#_Toc527840151)

[Powder X-ray diffraction (PXRD) 3](#_Toc527840152)

[N_2_ sorption 3](#_Toc527840153)

[UV 3](#_Toc527840154)

[Element analyses 3](#_Toc527840155)

[S3. Materials synthesis 3](#_Toc527840156)

[Synthesis of UiO-67-bpdc and UiO-67-bpydc 3](#_Toc527840157)

[Synthesis of DUT-5 4](#_Toc527840158)

[Synthesis of [Cp*Ir(bpydc)(Cl)]Cl 4](#_Toc527840159)

[In situ NMR-Scale Solvent-Assisted Ligand Exchange (SALE) 4](#_Toc527840160)

[Laboratory-Scale Solvent-Assisted Ligand Exchange (SALE) 5](#_Toc527840161)

[Postsynthetic modification 5](#_Toc527840162)

[S4. Characterization Data for Synthesized and Modified Materials 6](#_Toc527840163)

[Powder X-ray Diffraction (PXRD) Analysis 6](#_Toc527840164)

[Inductively-coupled plasma optical emission spectroscopy (ICP-OES) analysis 7](#_Toc527840165)

[N_2_ sorption and desorption isotherms 8](#_Toc527840166)

[Reproducibility 9](#_Toc527840167)

[Initial Stage of SALE on a Semilogarithmic Scale 9](#_Toc527840168)

[Effect of Ligand Loading on SALE 10](#_Toc527840169)

[Converse reaction 10](#_Toc527840170)

[Specific surface area 11](#_Toc527840171)

[Appearance of bpydc_(frame)_ in the Liquid State in the Presence of Acetic Acid 12](#_Toc527840172)

[UV-visible spectroscopy 13](#_Toc527840173)

[S5. Raw in situ ^1^H NMR data used to generate Figures 2–6 14](#_Toc527840174)

## Materials

Unless otherwise described below, all starting materials and solvents were obtained from commercial suppliers (Alfa Aesar, Sigma-Aldrich, VWR, Solveco, Cortecnet) and used without further purification.

## Methods

### NMR

Details of the ^1^H NMR experiments are found in the Experimental section in the paper.

For initial experiments to detect adsorption of linker onto UiO-67, a capillary containing 4,4-dimethyl-4-silapentane-1-sulfonic acid (DSS) in D_2_O was used as an internal standard.

Normalized concentration:

The number of moles of linkers in solution were normalised using the sum of the peak integrals of the linkers from the first recorded experiment and the known amount of linker_(sol)_. To account for the different linker_(sol)_:linker_(frame)_ ratios, the moles of linker were normalized by the known moles of the linker_(frame)_.

Solid-state: Proton nuclear magnetic resonance (^1^H NMR) experiments were recorded on a 14.1 T (^1^H Larmor frequency 600 MHz) Bruker Avance III spectrometer equipped with a 4-mm triple resonance probe head. Samples were packed in a zirconia rotor and spun at 14 kHz. The ^1^H chemical shift was referenced externally to Si(CH_3_)_4_. The relaxation delay was set to 20 s.

### Powder X-ray diffraction (PXRD)

Powder X-ray diffraction was performed on a PANalytical X’Pert PRO MPD with Cu-Kα_1_ radiation (λ = 1.542 Å). The sample was spread on a 20-mm Si wafer for measurement.

### N_2_ sorption

Surface area and porosity of the activated materials were investigated using N_2_ sorption isotherms, which were measured at –196 ºC on a Micromeritics ASAP 2020 system. Prior to measurement, the samples were degassed for 6 h at 120 °C.

### UV

UV-visible spectra were measured over the range 250–800 nm on a Perkin–Elmer Lambda19 spectrometer. The sample was supported on a glass slide and measured against anhydrous BaSO_4_ as the blank.

### Element analyses

Inductively-coupled plasma–optical emission spectroscopy (ICP–OES) was performed at MEDAC Ltd (Surrey, UK).

## Materials synthesis

### Synthesis of UiO-67-bpdc and UiO-67-bpydc

The synthesis of UiO-67 was based on the method proposed by Fei et al. [1]. A synthesis batch consisted of six glass vials prepared using identical starting materials and procedure. Anhydrous ZrCl_4_ (24.5 mg, 0.105 mmol), H_2_bpdc (biphenyl-4,4′-dicarboxylic acid) or H_2_bpydc (0.105 mmol) and glacial acetic acid (210 mg, 3.5 mmol) were combined in a 20-mL glass vial with 4 mL anhydrous dimethylformamide (DMF). The mixture was sonicated for 15 min and then incubated at 120 °C for 24 h. After cooling to room temperature, the solid products were merged into two falcon tubes and centrifuged at 9000 rpm for 15 min at room temperature before the solvent was decanted. A series of washes were then performed. Between each washing, the suspension was centrifuged at 9000 rpm for 15 min, and the solvent was decanted and replaced with fresh solvent. The solid was first sonicated 3× in fresh lukewarm DMF (60 mL) for 30 min, then soaked overnight in fresh ethanol (99.5%, 60 mL) under gentle stirring, and finally twice more with fresh ethanol for 3 h each time. The final solid product was dried under vacuum for 48 h. The yield of UiO-67-bpdc was 178.6 mg, corresponding to 80% yield based on Zr, and that of UiO-67-bpydc was 96.8 mg, a 65% yield based on Zr. In both cases, a visible amount of product was lost during the activation procedure.

### Synthesis of DUT-5

The synthesis of DUT-5 was based on the method proposed by Senkovska et al. [2]. A synthesis batch consisted of three vials prepared using identical starting materials and procedure. AlCl_3_·6H_2_O (64.6 mg, 0.268 mmol), H_2_bpdc (65 mg, 0.268 mmol) were combined in a 20-mL microwave reaction vial (VWR) with 7.5 mL anhydrous DMF, 48 µL deionized water and benzoic acid (327 mg, 2.68 mmol). The mixture was sonicated for 15 min and then heated at 120 °C in an oil bath for 24 h while stirring. After a few minutes, the mixture became a clear solution, then gradually turned into a milky suspension. After cooling to room temperature, the solid was merged into two falcon tubes and centrifuged at 9000 rpm for 15 min at room temperature before the solvent was decanted. The solid product washed according to the same procedure used in the synthesis of UiO-67 (vide supra), then dried under vacuum for 48 h to give 176 mg dry powder, corresponding to a 77% yield based on Al. A noticeable amount of product was lost during the activation procedure.

### Synthesis of [Cp*Ir(bpydc)(Cl)]Cl

[Cp*Ir(bpydc)Cl]Cl metallolinker was synthesized as described in reference [3].

### In situ NMR-Scale Solvent-Assisted Ligand Exchange (SALE)

The solution containing the dissolved linker was prepared in a vial. Separately, the desired amount of UiO67-bpdc or UiO67-bpydc was weighed directly into the NMR tube. 1 ml of the linker solution was added to the NMR tube, which was then sonicated for a couple of minutes before being inserted into the preheated spectrometer. The spectrometer temperature was calibrated using the chemical shifts of methanol.[4]

### Laboratory-Scale Solvent-Assisted Ligand Exchange (SALE)

Measurement of liquid-state ^1^H NMR spectra from ex situ experiments

H_2_bpdc (6.8 mg) was dissolved in 10 mL DMSO-*d*_6_ and H_2_bpydc (4.62 mg) in 7 mL DMSO-*d*_6_. A magnetic stir bar was added and the solvent was heated with UiO-67-bpydc (13 mg) or UiO-67-bpdc (11.7 mg) (as-synthesized or ground in an agate mortar and pestle). The flask was lowered into an oil bath that had been preheated to 57 °C and the mixture was allowed to stir for 96 h at that temperature. Aliquots of 0.1 or 0.2 mL were transferred into an NMR tube after 1, 2, 6, 24, and 28 h, and diluted to 0.6 mL with further DMSO-*d*_6_. ^1^H NMR spectra was recorded at room temperature.

Ex situ experiments to analyze the composition of linker in UiO-67-(bpdc/bpydc)

In DMSO: 23.3 mg H_2_bypdc was dissolved in 35.3 mL DMSO. A magnetic stir bar was added and the solvent was heated with 60 mg UiO-67-bpdc (ground in an agate mortar and pestle). The flask was lowered into an oil bath that had been preheated to 60 °C and the mixture was allowed to stir for 24.5 h at that temperature. The mixture was allowed to cool to room temperature and filtered, and the solid was washed several times with fresh ethanol. The final solid product was dried under vacuum at room temperature and characterized by PXRD, N_2_ adsorption and solid-state NMR.

In DMF: 19.94 mg H_2_bpydc was dissolved in 33.3 mL DMF. A magnetic stir bar was added and the solvent was heated with 50 mg UiO-67-bpdc (ground in an agate mortar and pestle). The flask was lowered into an oil bath that had been preheated to 60 °C and the mixture was allowed to stir for 18 h at that temperature. The mixture was allowed to cool to room temperature and filtered, and the solid was washed with fresh ethanol several times. The final solid product was dried under vacuum at room temperature and characterized by PXRD, N_2_ adsorption and solid-state NMR.

### Postsynthetic modification

UiO-67-bpydc (8.9 mg) and 1,2-diiodoethane (74.8 mg) were weighed into an NMR tube that was equipped with a Teflon closure. Deuterated tetrahydrofuran (THF-*d*_8_, 0.7 mL) was added, and the sample tube was sealed and lowered into the NMR spectrometer, which had been preheated to 55 °C, and spun at 20 Hz. NMR spectra were recorded according to the procedure described above. After six days, the sample was removed from the spectrometer and allowed to cool to room temperature. The solid was filtered and washed multiple times with non-deuterated THF before its UV-visible spectrum was recorded.

## Characterization Data for Synthesized and Modified Materials

### Powder X-ray Diffraction (PXRD) Analysis


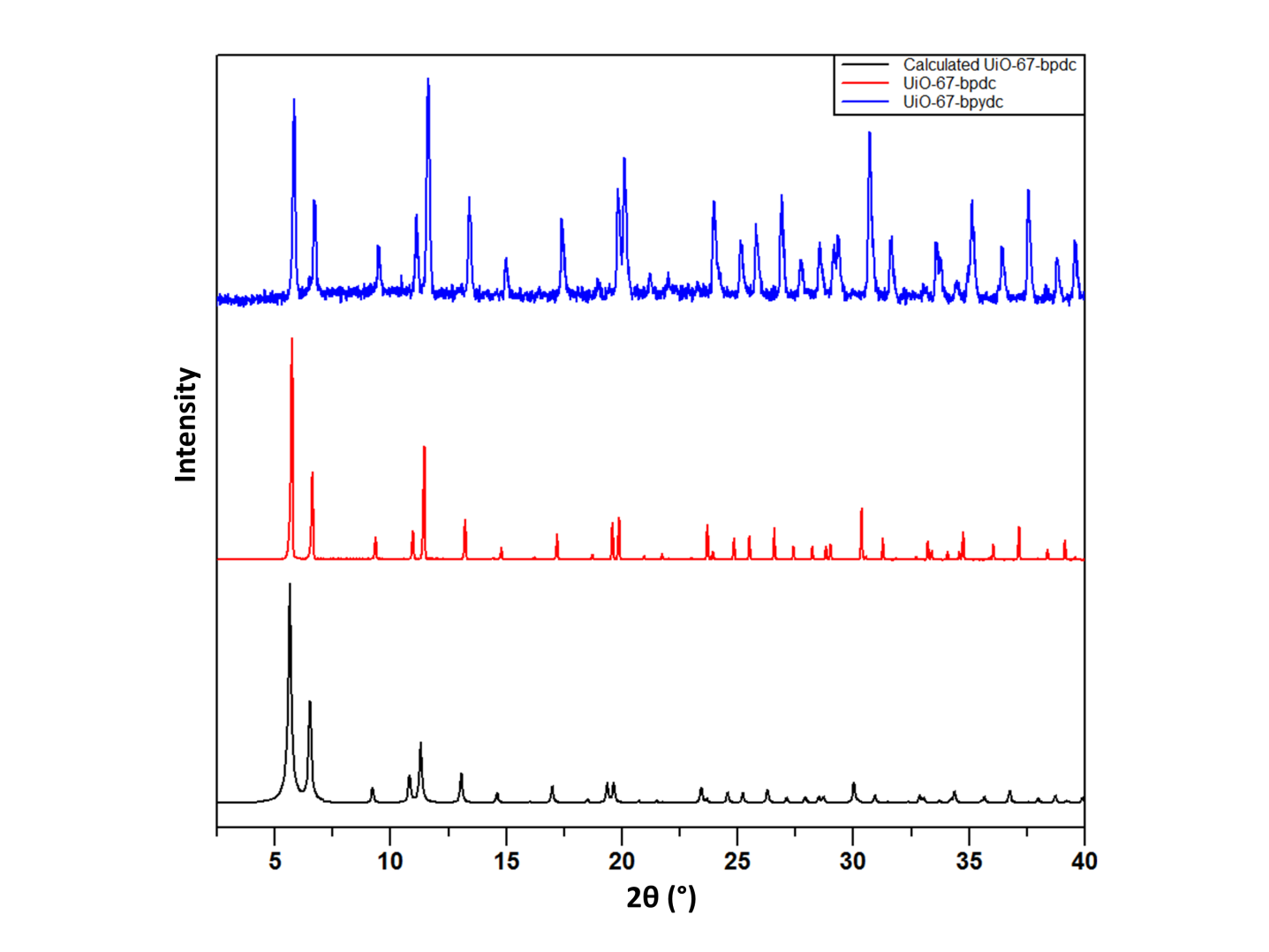


**Figure S1**. PXRD patterns of calculated UiO-67-bpdc (black) as well as of synthesized UiO-67-bpdc (red) and UiO-67-bpydc (blue).

**Figure S2**. PXRD patterns of synthesized UiO-67-bpdc (red), of UiO-67-bpdc after ex situ SALE with bpydc_(sol)_ in DMF (brown) or DMSO (purple), and UiO-67-bpdc after soaking in MeOH (green).


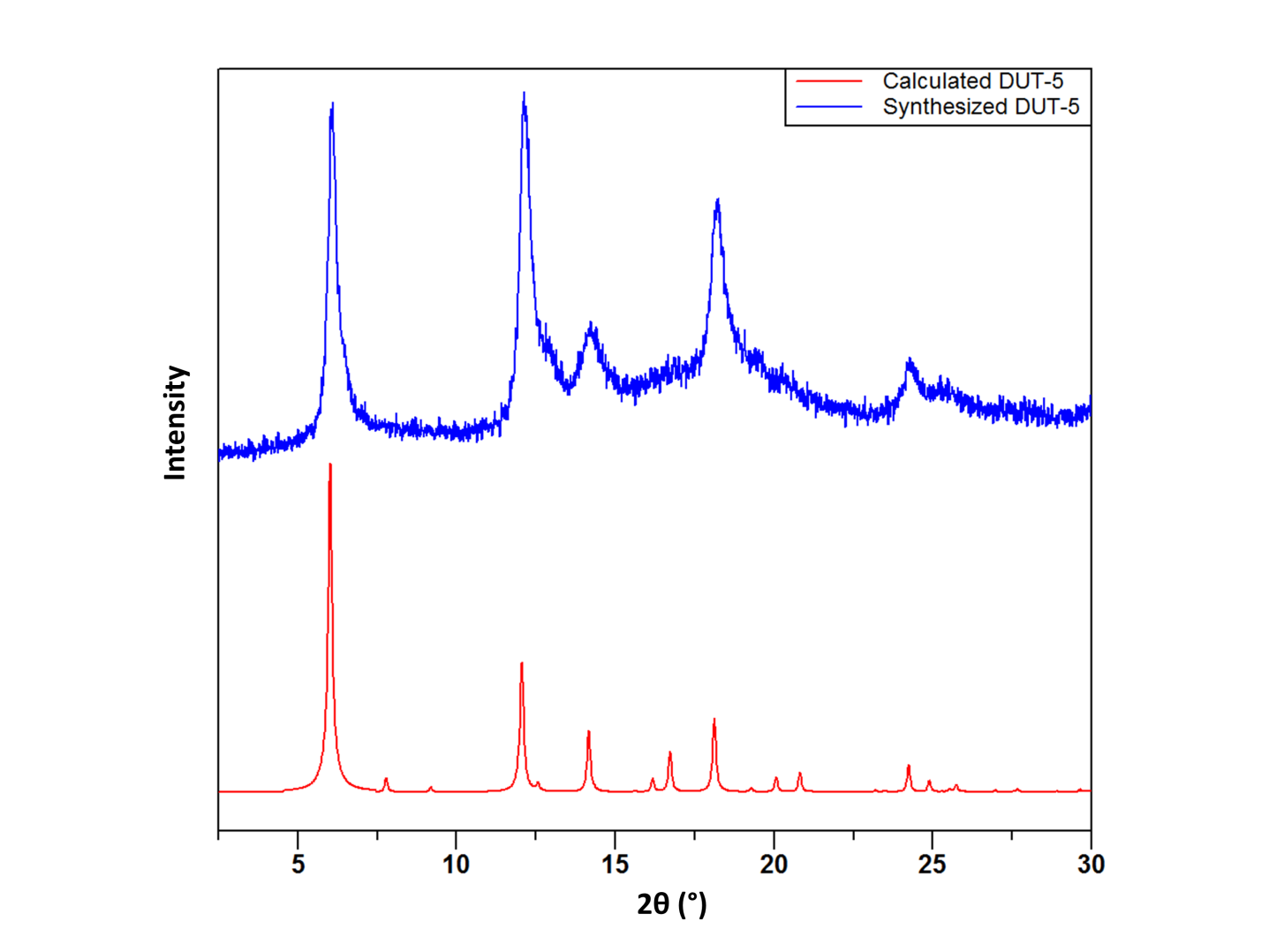


**Figure S3**. PXRD patterns calculated (red) and synthesized DUT-5 (blue).

### Inductively-coupled plasma optical emission spectroscopy (ICP-OES) analysis

The composition analysis of UiO-67-bpdc that was used as the starting material for the linker exchange and dissolution experiments was analyzed by ICP–OES. The as-synthesized UiO-67-bpdc has a formula of Zr_6_O_4_(OH)_4_(C_14_H_8_O_4_)_5.85_(C_2_H_3_O_2_)_0.49_ with a negligible (<0.10 wt%) N content, and therefore a negligible amount of DMF. The empirical formula of UiO-67-bpdc is Zr_6_O_4_(OH)_4_(C_14_H_8_O_4_)_6_ [1, 5]. Thus, a small fraction of the bpdc linkers in the theoretical UiO-67-bpdc structure are replaced by acetate in the synthesized material, and some additional acetate is present, but no free bpdc linker remains.

### N_2_ sorption and desorption isotherms

| 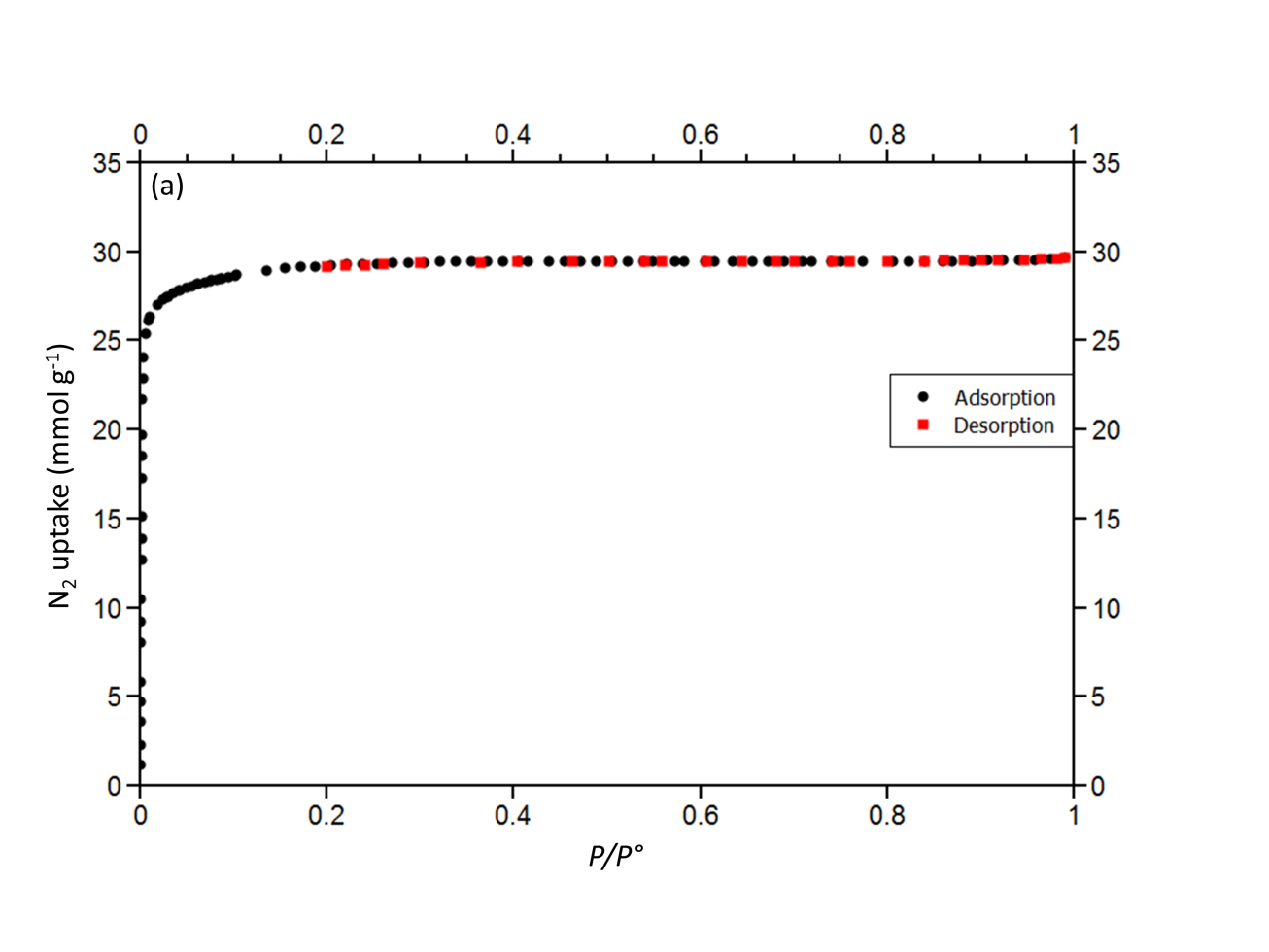 | 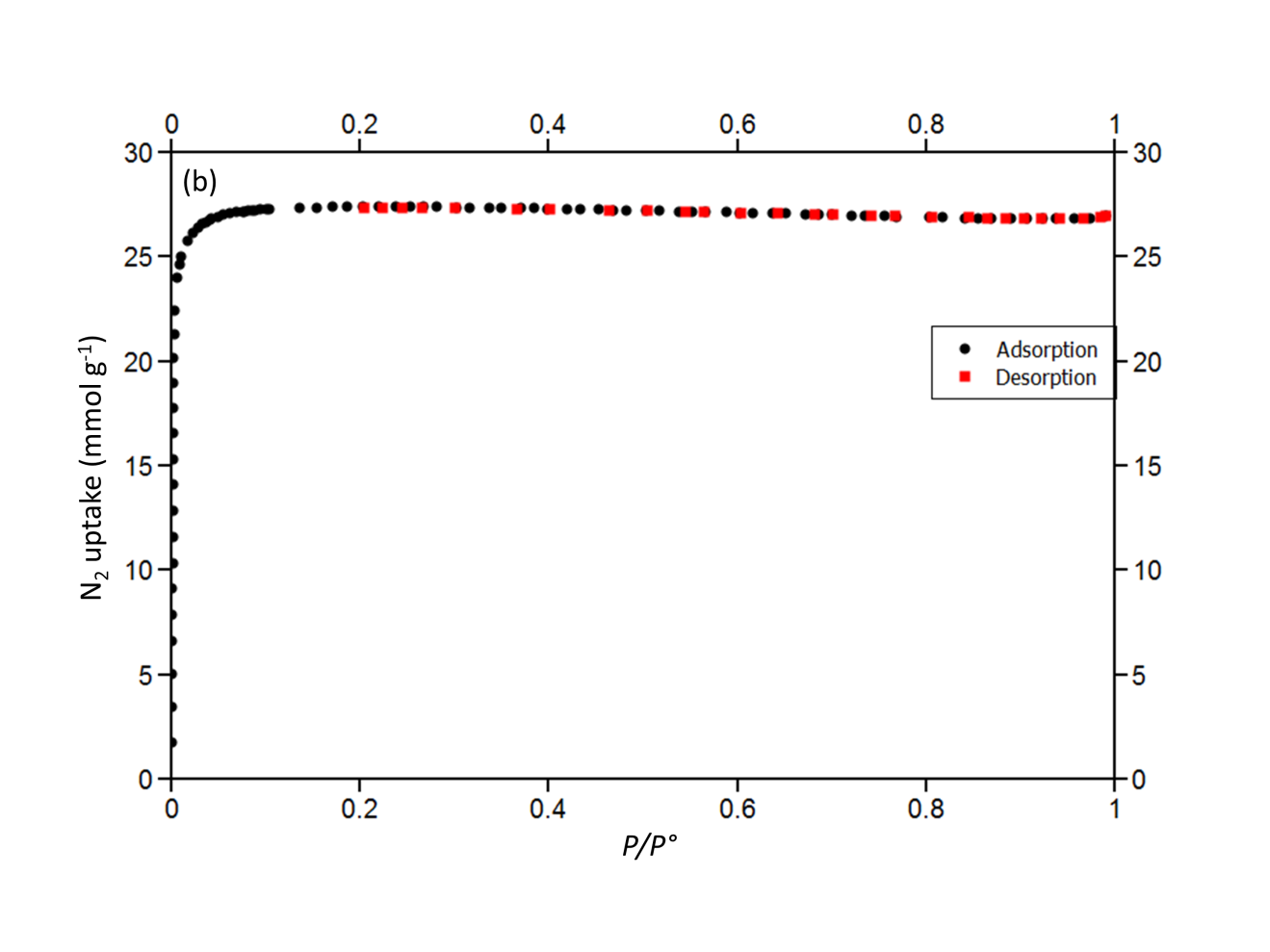 |
| --- | --- |
| 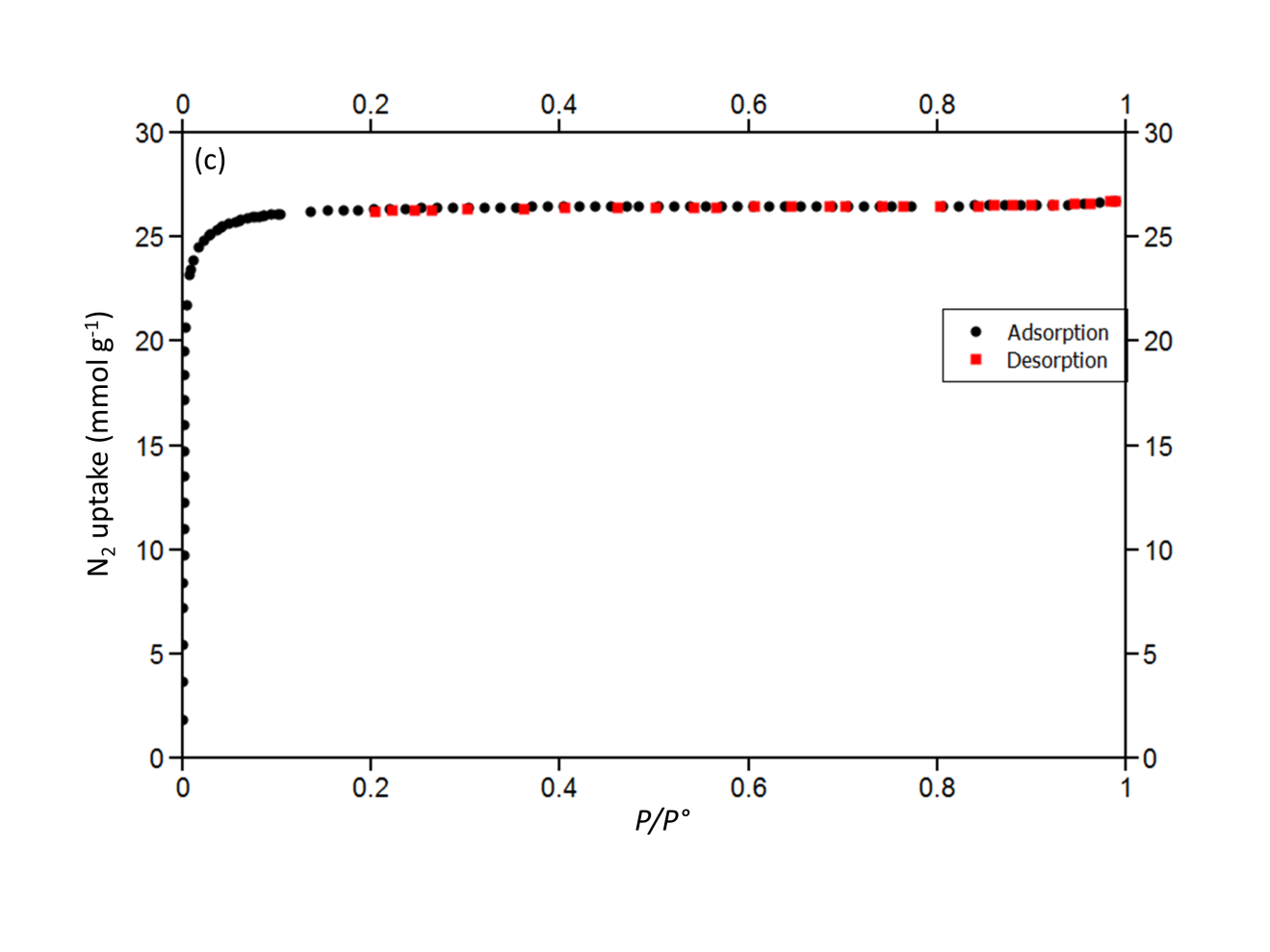 |  |
| **Figure S4**. N_2_ adsorption and desorption isotherms of (a) UiO-67-bpdc and of (b) UiO-67-bpdc after SALE (see S3) for bpydc in DMF and (c) in DMSO. | |

### Reproducibility

**Figure S5**. The concentrations of linkers in the liquid state during the reaction of UiO-67-bpdc and bpydc_(sol)_ in DMF-*d*_7_ at 57 ºC, as functions of time. Two independent measurements are displayed.

### Initial Stage of SALE on a Semilogarithmic Scale

**Figure S6**. Natural logarithm of the normalized concentration of linkers (see S2) in the liquid state of SALE as a function of time, during the initial portion of the reaction, as calculated from ^1^H NMR spectroscopy. Reactions were performed at 57 ºC.

### Effect of Ligand Loading on SALE


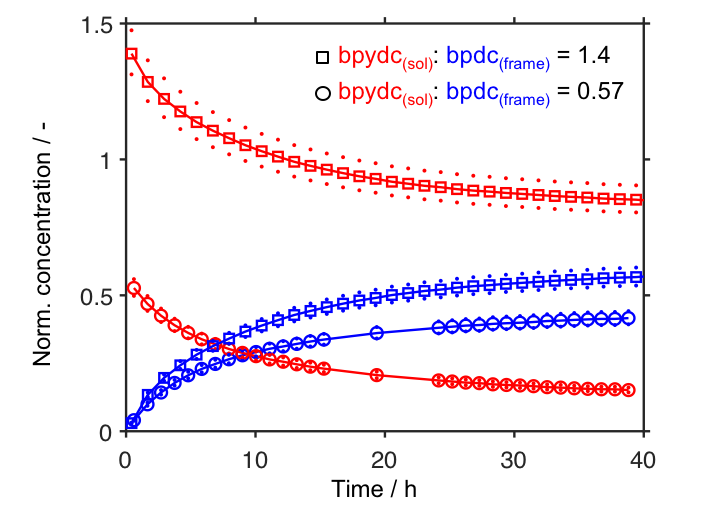


**Figure S7**. Normalized concentrations of linkers (see S2) for in the liquid state during the reaction of UiO-67-bpdc and bpydc_(sol)_ as functions of time. UiO-67-bpdc (1.7 mg) was heated H_2_bpydc in 1.0 mL DMSO-*d*_6_ at 57 ºC and the reaction was monitored using in situ ^1^H NMR spectroscopy. Dots represent the spread of the normalized concentrations calculated from the mass of MOFs and the uncertainty of the balance (0.1 mg).

### Converse reaction


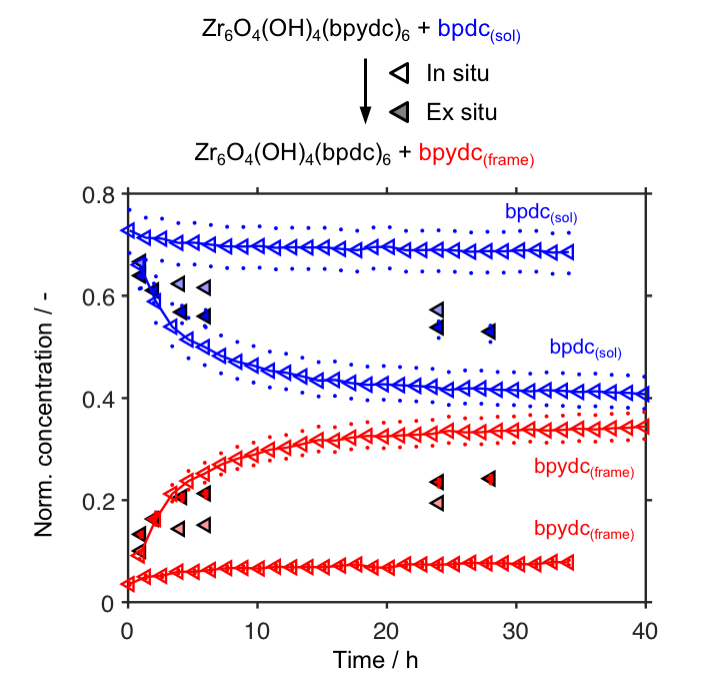


**Figure S8**. Normalized concentrations of linkers (see S2) in the liquid state of SALE as functions of time, as calculated from ^1^H NMR spectroscopy. Reactions, UiO-67-bpydc + bpdc_(sol)_ were performed in DMSO-*d*_6_ at 57 °C. Dots represent the spread of the normalized concentrations calculated from the mass of MOF present and the uncertainty of the balance (0.1 mg).

**Figure S9**. Solid-state ^1^H NMR spectra recorded for ex situ SALE (see S3) for UiO-67-bpdc in DMF. Bpydc_(sol)_ and bpdc_(frame)_ integrals were estimated by deconvolution of the peaks using the combination of four Lorentzian lines.

**Figure S10**. Solid-state ^1^H NMR spectra recorded for ex situ SALE (see S3) for UiO-67-bpdc in DMF. Bpydc_(sol)_ and bpdc_(frame)_ integrals were estimated by deconvolution of the peaks using the combination of four Lorentzian lines.

### Specific surface area

Table S1. Specific surface areas and pore volume of UiO-67-bpdc before and after ex situ SALE in DMF and DMSO as solvents.

|  | BET surface area (m^2^ g^-1^) | Langmuir surface area (m^2^ g^-1^) | Pore volume  (cm^3^ g^-1^) |
| --- | --- | --- | --- |
| UiO-67-bpdc | 2004^a^ | 2905^b^ | 1.03 |
| Ex situ SALE (bpydc) DMF | 1868 ^a^ | 2681^c^ | 0.93 |
| Ex situ SALE (bpydc) DMSO | 1702 ^a^ | 2593^d^ | 0.93 |

^a^ *p*/*p*^0^ from 0.061 to 0.013; ^b^ *p*/*p*^0^ from 0.048 to 0.152; ^c^ *p*/*p*^0^ from 0.048 to 0.150; ^d^ *p*/*p*^0^ is from 0.049 to 0.155

### Appearance of bpydc_(frame)_ in the Liquid State in the Presence of Acetic Acid


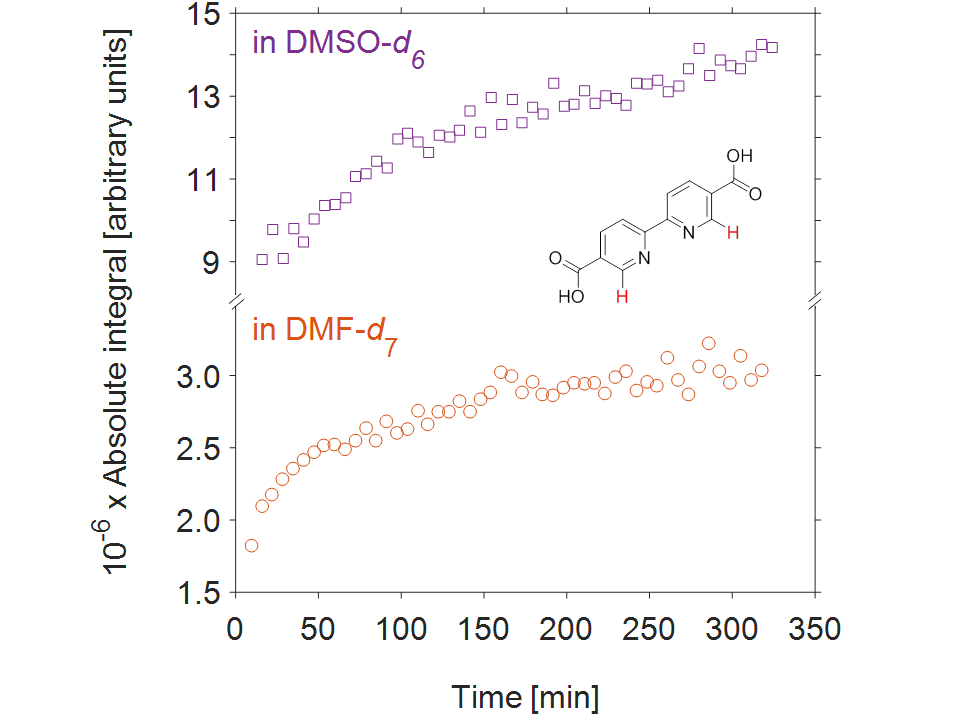


**Figure S11**. Appearance of bpydc_(frame)_ from UiO-67-bpydc in DMSO-*d*_6_ or DMF-*d*_7_ following the addition of 1 μL acetic acid. The ^1^H NMR signal at 9.3 ppm, assigned to the indicated protons in bpydc (in red), was monitored.

**Figure S12**. (a) Liquid-state ^1^H NMR spectra recorded after heating UiO-67-bpdc in CD_3_OD. DMF is an impurity derived from the synthesis of the MOF. (b) Absolute ^1^H integral for peak 1 versus time.

### UV-visible spectroscopy

**Figure S13**. UV-visible spectrum of solid UiO-67-bpydc after reaction with ICH_2_CH_2_I in THF-*d*_8_ at 55 °C under in situ NMR spectroscopy conditions, then filtration and washing with THF.

*Viscosity in C_6_D_6_/DMSO-d_6_ and DMSO-d_6_*

Here we assume that the viscosities of the solvents and their mixtures are unaffected by deuteration. The viscosities *η* of benzene/dimethylsulfoxide mixtures (non-deuterated solvents) have been measured over the temperature range 25–35 °C [6]. The diffusion coefficient *D* is proportional to the inverse of the viscosity *η*, so using an Arrhenius model for the temperature dependence of *D,*

| ln(*η*^–1^) = a*T*^–1^ + c | (1) |
| --- | --- |

where a and c are constants that can be determined from a plot of ln(*η*^–1^) vs. *T*^–1^, as in Figure S10 for DMSO–benzene mixtures having 100, 40.7, and 0 mol% DMSO.

**Figure S14**. Relationship between ln(*η*^–1^) and *T*^–1^ for DMSO, benzene, and a mixture of the two, over the temperature range 25–35°C. Viscosity data is from reference [6].

From linear fits of the data in Figure S8, viscosities of 0.943, 1.75, and 2.59 can be calculated for DMSO, 40.7 mol% DMSO in benzene, and benzene, respectively, at 60 °C. Therefore, the ratio of diffusion coefficients for a given molecule in 40.7% DMSO in benzene and in benzene is expected to be 1.86 at this temperature.

## Raw in situ ^1^H NMR data used to generate Figures 2–6

**Figure S15**. Raw data used to generate Figure 2. Stacked ^1^H NMR spectra versus time for the solvent-assisted linker exchange reaction of UiO-67-bpdc with bpydc_(sol)_ in DMSO-*d*_6_ at 57 °C.

**Figure S16**. Raw data used to generate Figure 2. Stacked ^1^H NMR spectra versus time for the solvent-assisted linker exchange reaction of UiO-67-bpdc with bpydc_(sol)_ in DMF-*d*_7_ at 57 °C.

**Figure S17**. Raw data used to generate Figure 3. Stacked ^1^H NMR spectra versus time for the solvent-assisted linker exchange reaction of UiO-67-bpdc with Ir-bpydc_(sol)_ in CD_3_OD at 57 °C (linker_(sol)_:linker_(frame)_ ratio = 0.41 by mole).

**Figure S18**. Raw data used to generate Figure 3. Stacked ^1^H NMR spectra versus time for the solvent-assisted linker exchange reaction of UiO-67-bpdc with Ir-bpydc_(sol)_ in CD_3_OD at 57 °C (linker_(sol)_:linker_(frame)_ ratio = 0.82 by mole).

**Figure S19**. Raw data used to generate Figure 5c. Stacked ^1^H NMR spectra versus time for the dissolution of UiO-67-bpdc in DMSO-*d*_6_ at 57 °C.

**Figure S20**. Raw data used to generate Figure 5c. Stacked ^1^H NMR spectra versus time for the dissolution of UiO-67-bpydc in DMSO-*d*_6_ at 57 °C.

**Figure S21**. Raw data used to generate Figure 6. Stacked ^1^H NMR spectra versus time for the reaction of UiO-67-bpydc and I(CH_2_)_2_I in THF-*d*_8_ at 55 °C.

**References**

[1] Fei, H.; Cohen, S. M. *Chem. Commun.* **2014**, *50*, 4810.

[2] Senkovska, I.; Hoffmann, F.; Fröba, M.; Getzschmann, J.; Böhlmann, W.; Kaskel, S. *Microporous Mesoporous Mater.* **2009**, *122*, 93.

[3] Platero-Prats, A. E.; Bermejo Gómez, A.; Samain, L.; Zou X.; Martín-Matute, B. *Chem. Eur. J.*, 2014, **21**, 861–866.

[4] Raiford, D. S.; Fisk, C. L.; Becker, E. D.; *Anal. Chem.*, **1979**, *51,* 2050.

[5] Ko, N.; Hong, J.; Sung, S.; Cordova, K.E.; Park, H.J.; Yang J.K.; Kim J. *Dalton Trans*. **2015**, *44*, 2047.

[6] Aralaguppi, M.I.; Aminabhavi, T.M.; Harogoppad, S.B.; Balundgi. R.H. *J. Chem. Eng. Data* **1992**, *37*, 298.
